# Child, Teacher and Parent Perceptions of the FRIENDS Classroom-Based Universal Anxiety Prevention Programme: A Qualitative Study

**DOI:** 10.1007/s12310-016-9187-y

**Published:** 2016-03-12

**Authors:** Elena Skryabina, Joanna Morris, Danielle Byrne, Nicola Harkin, Sarah Rook, Paul Stallard

**Affiliations:** Department for Health, University of Bath, Bath, BA2 7AY UK

**Keywords:** Anxiety, Prevention, Schools, CBT, Children, FRIENDS

## Abstract

School-based mental health prevention programmes can be effective but their adoption within schools will depend on their social acceptability. We report a qualitative evaluation summarising the views of children (115), parents (20) and school staff (47) about a universal school-based anxiety prevention programme FRIENDS. This study was conducted as part of a large scale randomised controlled trial (*n* = 1362) involving 40 schools in the UK providing primary education to children aged 7–11. Reported overall experience of the programme was very positive, with all three major components of the cognitive behaviour therapy programme (emotional, cognitive, and behavioural) being accepted well and understood by children. The programme was considered to be enjoyable and valuable in teaching children important skills, particularly emotional regulation and coping. Children provided examples of using the skills learned during FRIENDS to manage their emotions and solve problems. However, teachers were concerned that the programme overlapped with the current school curriculum, required additional time and almost half were unable to identify any tangible changes in the children’s behaviour. Whilst this paper provides evidence to support the social validity of the FRIENDS anxiety prevention programme, the concerns raised by teachers question the longer-term sustainability of the programme.

## Introduction

Anxiety is the most prevalent psychiatric disorder affecting around 10 % of children and up to 20 % of adolescents (Essau, Conradt, Sasagawa, & Ollendick, 2012). Childhood anxiety can prevent children from participating in age-related activities and can have adverse effects on education, and social and psychological development (Barrett, Lock, & Farrell, 2005). The onset of anxiety often begins early in life, but the course is commonly chronic and recurrent (Costello, Mustillo, Erkan, Keller, & Angold, 2003). If left untreated, anxiety increases the risk of developing other problems in young adulthood including depression and substance abuse and is associated with unemployment in later life (Kim-Cohen et al., 2003; Woodward & Fergusson, 2001).

Effective psychological treatments, especially cognitive behavioural therapy (CBT), are available for children with anxiety disorders (Reynolds, Wilson, Austin, & Hooper, 2012). However, comparatively few children with anxiety disorders receive specialist help with most remaining unidentified and untreated (Essau, 2007; Merikangas et al., 2010). This, along with the limited availability of specialist mental health services, has led to interest in early intervention and prevention programmes (Farrell & Barrett, 2007). Schools provide an accessible and convenient location to deliver mental health interventions, and they are increasingly expected to play a significant role in promoting and supporting the mental health of children (Department for Education and Skills, 2005).

Prevention programmes have been described as universal or targeted depending upon who receives them (Mrazek & Haggerty, 1994). In universal approaches, all of a population, e.g. whole school, receive the intervention regardless of risk factors or perceived symptoms. In targeted approaches interventions are focused upon children who have emerging symptoms or are at increased risk of developing problems. Targeted interventions typically produce larger effect sizes and are a more focused use of limited financial resources, whereas universal interventions are less stigmatising and have the advantage of reaching larger numbers of children (Ahlen, Breitholtz, Barrett, & Gallegos, 2012; Barrett, 2001; Barrett, Farrell, Ollendick, & Dadds, 2006).

Over the past decade, a number of school-based anxiety prevention programmes have been developed, with programmes based upon cognitive behaviour therapy being identified as the most effective (Neil & Christensen, 2009). One particular CBT programme, ‘FRIENDS for Life’ (Barrett, 2004), developed and initially evaluated in Australia appears to be particularly promising (Fisak, Richard, & Mann, 2011). There are three versions of the programme matched to the age and developmental level of the child. Fun FRIENDS is for younger children (aged 4–7), and there are versions of FRIENDS for children (aged 8–11) and youth (aged 12–16). Children have an attractive workbook and through a series of fun and enjoyable activities learn to identify and manage their anxious feelings, to develop more helpful (anxiety reducing) ways of thinking and to face and overcome fears and challenges rather than avoid them (see Table 1 for definition of the acronym).

**Table 1:** FRIENDS acronym definition

Acronym	Meaning
**F**	**F**eelings
**R**	**R**emember to **R**elax
**I**	**I** can do it! I can try my best!
**E**	**E**xplore solutions and coping step plan
**N**	**N**ow reward yourself
**D**	**D**on’t forget to practise
**S**	**S**mile! **S**tay calm for life!

FRIENDS is effective as a targeted intervention (Bernstein, Layne, Egan, & Tennison, 2005; Dadds, Holland, Laurens, Barrett, & Spence, 1999; Lowry-Webster, Barrett, & Dadds, 2001) and when delivered to whole classes of children as a universal intervention (Barrett et al., 2005; Barrett & Turner, 2001). The effects of FRIENDS have been maintained at 3 year follow-up (Barrett et al., 2006) although recent implementation trials outside of Australia have found mixed results. Targeted and universal studies conducted in Canada found no effect when FRIENDS was delivered by class teachers (Miller et al., 2011). The lack of effectiveness of FRIENDS when delivered by school staff was also found in a large UK randomised trial; however, FRIENDS demonstrated significant anxiety prevention effects when delivered by health staff from outside of the school (Stallard et al., 2014).

In addition to who delivers the programme, other potentially important factors include student engagement, relevance of content, attractiveness of materials and teacher and pupil views about usefulness (Neil & Christensen, 2009; Rapee et al., 2006). Qualitative evaluations may provide a useful insight into these factors but to date no qualitative analysis of FRIENDS exploring these different perspectives have been reported (Barrett, Shortt, Fox, & Wescombe, 2001). Establishing social validity using robust qualitative methodology such as thematic analysis (Braun & Clarke, 2006) is important if anxiety prevention programmes are to be adopted by schools. Indeed, effective programmes may not be used unless they are acceptable and perceived as useful by children and school staff. This study addresses this issue and summarises the views of children, parents, and school staff who participated in a randomised controlled trial of FRIENDS.

## Method

### Design

The study was undertaken as part of a large randomised controlled trial PACES (Preventing Anxiety in Children through Education in Schools) (Stallard et al., 2012). It is a qualitative study using semi-structured individual interviews or focus groups, conducted with three groups of participants: children, teacher and parents. Data were analysed using thematic analysis.

### Context: Preventing Anxiety in Children through Education in Schools (PACES)

PACES is a large UK randomised controlled trial comparing FRIENDS delivered as a universal intervention by health and school staff with usual school provision of Personal, Social and Health Education (PSHE) lessons (Stallard et al., 2012). A total of 1362 children aged 9–10 attending 40 schools in the South West of England took part in the trial with 1257 (92.3 %) being reassessed at 12 months. The interventions were delivered over the academic year of 2011/2012. FRIENDS was effective in reducing anxiety compared to usual school provision but only when delivered by health staff (Stallard et al., 2014).

### FRIENDS

FRIENDS is a CBT programme delivered in 45–60 min sessions over 9 consecutive weeks to whole classes of children. FRIENDS helps children develop emotional awareness and emotional regulation skills, to replace thoughts that increase anxiety with those that reduce and help to cope with anxiety, to manage anxious feelings with relaxation techniques, and to face challenges with problem solving skills. A summary of individual session is provided in Table 2.

**Table 2:** FRIENDS session plan

Session number	Content
Session 1	Introduction to FRIENDS
Session 2	Introduction to feelings
Session 3	The relationship between thoughts and feelings
Session 4	Emotional recognition, relaxation and how to feel good
Session 5	Developing positive self-talk
Session 6	Challenging negative/unhelpful thoughts
Session 7	Developing problem solving skills

FRIENDS was delivered to classes by either 14 trained school staff (e.g. classroom teachers, special educational needs co-ordinators, teaching assistants and head teachers/principals) or 11 health staff external to the school. Health staff had at least an undergraduate university degree in a relevant discipline (e.g. psychology, art therapy) or an appropriate professional background (e.g. nursing, educational) and experience of working with children or young people.

All FRIENDS leaders attended a 2-day training event provided by accredited FRIENDS trainers to familiarise them with the nature, extent and presentation of anxiety and depression in children and the CBT model. Participants worked through each of the FRIENDS sessions and practiced the exercises to familiarise themselves with the materials and key learning points.

Parents were sent a brief overview of the FRIENDS programme and were invited to attend an information meeting at their child’s school. The aim of the meeting was to give parents an overview of the programme, the CBT model and the skills the children would learn so that they could support their child’s use of skills at home.

### Participants

#### School Staff

Qualitative interviews were undertaken with staff from all of the 28 schools who received the FRIENDS programme. Feedback was obtained from 15 Head teachers/school principals, 24 class teachers and 8 PSHE co-ordinators who were responsible for the personal, social and emotional health curriculum provided by the school. Those interviewed had direct experience either leading or supporting the delivery of the FRIENDS programme in their school.

#### Children Who Received FRIENDS

During the academic year 2011/2012 FRIENDS was delivered to 12 schools in the first academic term, 11 schools in the second term and 5 schools in the third term. After completing the programme children were asked to volunteer to participate in focus groups to explore their experience of FRIENDS in more detail. We planned to interview 10 % of children, a sample size expected to be sufficient to allow saturation of dominant themes. By the end of the second term we had interviewed 12 % of our total cohort, 115 children (51 boys and 64 girls). The children attended 19 of the 28 schools who received FRIENDS, 10 schools received FRIENDS delivered by health staff and 9 by school staff. Child ethnicity and socio-economic status were not recorded, but the overall trial sample was largely dominated by children from British white background (94 %) and high affluence (Stallard et al., 2014).

#### Parents of Children Who Received FRIENDS

Twenty parents, mostly mothers, were randomly selected from a group of 308 who volunteered to take part in detailed interviews and were interviewed individually. Most parents had either a diploma from a further education college (40 %), a bachelor’s degree from a University (20 %) or postgraduate qualification such as a master’s degree or doctorate (10 %). The remaining 30 % had school level qualifications, either General Certificate of Secondary Education (15 %) completed at the age of 16, or a General Certificate of Education Advanced level (15 %) completed at the age 18. 65 % of parents were in part-time employment, 10 % employed full-time, and the remaining 25 % were unemployed. Average age of the interviewed parents was 42 years (min 28 and max 51).

Feedback was gathered separately from the children, parents, and school staff a few weeks after completing the programme. Interviews and focus groups were conducted between January and July 2012.

### Ethical Approval

Ethical review and approval for this study was provided by the University of Bath School for Health Ethics Committee. Children, parents and teachers gave their personal consent to take part in the interviews or focus groups.

### Data Collection

Children, parents and teachers participated in semi-structured interviews or focus groups to obtain feedback about their experience and perceptions of the FRIENDS programme. Participants were asked for their overall views about the programme, their most positive and negative experiences, the skills the children learned, programme contribution to the school PSHE curriculum and how the children had benefited. In addition, parents were asked about changes in their children’s mood and anxiety, general behaviour, overall confidence, friendship, engagement in out of school social and recreational activities and educational progress.

Interviews and focus groups were conducted by ES (Project Manager) who was not directly involved in programme delivery. All were digitally audio-recorded and were supplemented with additional notes.

### Data Analysis

All recordings were transcribed verbatim by four researchers using NVIVO 10 software (NVIVO, 2012). NVIVO is a qualitative data analysis computer software package that helps to classify, organise and analyse text-based information. The software facilitates accurate and transparent analysis of the data whilst providing an auditable count of who said what and when, thereby providing a reliable, general picture of the data.

The transcribed data were subjected to thematic analysis using the following approach: (1) becoming familiar with the data; (2) generating initial codes; (3) searching for themes; (4) reviewing themes and (5) defining and naming themes (Braun & Clarke, 2006). Initial coding was conducted inductively, i.e. by explicit or surface meaning of the data, without looking beyond what a participant said, and openly, i.e. without trying to fit into the pre-existing coding frame and allowing to form as many codes as felt appropriate. The same strategy was applied for coding data obtained from interviews and focus groups.

Coding reliability and validity was checked by asking two researchers independently to code three randomly selected transcripts, one for each category of respondents (children, teacher and parent) and then the inter-rater reliability was calculated using NVIVO 10 (NVIVO, 2012). The coding agreement was in the range of 79–100 %, indicating satisfactory agreement and consistency. The remaining data were coded by three researchers, using the generated framework, but with flexibility to expand it to fit the data to allow for open coding. Each of the three researchers completed coding data for one particular participant group.

Final codes were then analysed by four researchers independently to generate an initial framework for the themes and then reviewed by the researchers together to define the final themes. Further analysis identified six distinctive themes relating to: programme overview, programme content and delivery, FRIENDS workbook, positive aspects of the programme, programme benefits and continued use of skills. The themes were checked for internal consistency. Further analysis involved building detailed data maps and examining data prevalence.

## Results

The results summarised below highlight the most frequently endorsed views and are supported by appropriate quotes. The views of children, teachers and parents are given separately. An overview of the major themes is provided in Table 3.

**Table 3:** Summary of main themes

Main theme	Key points		
	Teacher (47)	Child (115)	Parent (20)
Programme overview (overall impression)	Good programme Useful Valuable	Fun Helpful	Good programme Children enjoyed FRIENDS
Programme content and delivery	Too many strategies, not enough time Too much passive learning Sessions plan useful Overlap with PSHE lessons	Liked hands-on activities and group work Wanted longer and more sessions	
FRIENDS Workbook	Children liked having their own workbook Too much reading and writing	Children liked the visual elements of workbook	
Positive aspects of the programme	Red and green thoughts Relaxation/pizza massage Coping step plan Problem solving techniques	Coping step plan Relaxation Balloon challenge	
Programme benefit	Learned to express feelings Recognise feelings in others Equipped teachers with strategies and language	Awareness and management of emotions Improved relationships Sharing feelings Recognising feelings in others	Improved relationships Talk to peers more easily Calmer Increased confidence Awareness of others and own feelings Academic improvements
Continued use of skills	Red and green thoughts Relaxation techniques No changes noticed in children’s behaviour	Relaxation Coping step plan Red and green thoughts	Relaxation techniques Problem solving techniques Changes not attributed to the FRIENDS

### Children’s Views

Feedback was obtained from 115 children aged 9–10 from 19 schools. Focus groups involved 2–9 children.

#### Overall Impression

Children were positive about FRIENDS, finding the programme both ‘helpful’ and ‘fun’.

I found it quite helpful as well because it gave you like different steps to like stop worrying and stuff.

They showed particular enthusiasm for the activity-based element of the programme finding it a refreshing change from their usual lessons.

Um, I really liked the way they put educational stuff into fun games. And sort of made it, like the different side of learning.

#### Programme Content and Delivery

Most children commented positively about the hands-on activities and group work such as, role play, creative tasks and games, and many wanted more of these activities.

I liked the one where you put the book on your head with a piece of paper, you draw with a pencil and you had to draw things without you looking so it was like, you were confident and you could do it.

Children wanted more time for FRIENDS, either longer sessions or additional sessions. They felt there wasn’t enough time to complete the tasks within the time available. They wanted to work through all the activities within the work book, feeling some disappointment that only some activities had been chosen to work with.

…the lessons could have been longer so we had more time to do the work book.

Sometimes we read bits in the FRIENDS book and then we skipped a few pages…and then it didn’t help us a lot, because some things that we skipped, they looked like they would help us…

Children made some negative comments about the health staff who led or supported the delivery of FRIENDS. Children from one school commented that the facilitators needed to speak slower and others noted that the delivery style could have been more confident, enthusiastic and disciplined.

I thought the people that were teaching it could have been a bit more sure and confident with what they were teaching.

Several children reported a dislike for the reading and writing aspects of the programme and commented that they would have preferred more activities and games.

I thought there was quite a lot of reading from the book, I would like some more maybe hands-on activities….

#### FRIENDS Workbook

Although children had mixed reviews about the FRIENDS workbook, overall the comments were mostly positive. Children said the book was ‘good’, and found the visual elements such the images and colours attractive.

I really liked the workbooks, how they explained things in not really complicated detail and pictures were really good in them as well…

#### Most Positive Aspects of the Programme

The majority of children were positive about the ‘coping step plan’ (problem solving technique) in which they are taught to break a problem or a challenge into smaller, more manageable steps.

It’s boosted my confidence because with coping step plan it showed me how to build up to something big……

Children also talked positively about the relaxation skills and particularly enjoyed the practical techniques they were taught.

… It taught us like how to if, if we’re getting really worried and built up about something it just taught us how to relax and sort of calm down and deal with it… It’s really good because it helps you relax and it’s really fun too.

Another memorable task was The ‘Balloon Challenge’ (problem solving). This involved working in groups and using a structured six step process to solve a problem.

I think some of the activities in the groups that we had to get the balloon across the classroom, they were quite good because you got to construct a thing and you get to work as a team and I found that quite helpful, to let us work as a team and share our ideas.

#### Programme Benefits

Children identified a number of ways in which the programme helped them. The most frequent overarching themes related to ‘awareness and management of emotions’ and ‘relationships’.

It’s really helped me to keep my emotions under control and not be too dramatic or anything.

It’s helped me to control my feelings and my sister annoys me and I’ve learned how to control not getting angry with her.

In addition, they spoke of learning to ‘recognising feelings in others’ and ‘recognising how people feel, think and react differently’, all of which helped to develop stronger and more supportive relationships. Some children also thought it helped them share their feelings and become more social.

Because it helped me work with some people who I didn’t normally work with it helped me realise how different people react to different situations.

……normally if I am upset I talk to someone I trust and who I really know about it and tell them about my feelings and that really does help.

#### Continued Use of Skills

Many children shared examples of actively using strategies and skills they were taught within the programme. The ‘coping step plan’ was used often, especially when they were faced with new challenges or learning to master something such as a stage performance or swimming.

I know some way it helped me…’cause it helped me overcome my fear of getting in the water, ‘cause now I can swim 5 metres.

A number reported using ‘Relaxation’ Skills such as allowing oneself or others time to calm down. These were mostly commonly used when faced with arguments both within school and home settings.

…………if you just like sort of close your eyes and just try and think things through with your eyes closed and just try and relax you might understand ….other people’s points of view as well.

Um, my cousin was feeling a bit grumpy a couple of weeks ago and I just, I just knew by the way she was just looking, I just knew that OK I’d better give her some space.

Several children provided examples of how they had changed Red (unhelpful anxiety increasing) thoughts to Green (helpful anxiety reducing) thoughts. Children spoke of doing this in situations such as Martial Arts grading, within the classroom and when they felt angry.

Sometimes my Mum gets angry and I say in my head my mum’s thinking red thoughts and then I say to her, can you think a green thought and then I can just see her trying to think the green thoughts and it just really helps me because I know she’s listening to me.

### Teacher Views

47 members of school staff from all 28 intervention schools who had been directly involved with the FRIENDS programme, provided their feedback. This was obtained from 15 head teachers/principals, 24 class teachers and 8 PSHE co-ordinators.

#### Overall Impression

The majority of the comments were positive with most teachers reporting that FRIENDS was a good programme based on valuable ideas and concepts and which the children enjoyed and found useful.

Overall, I thought it was very valuable,… the message in particular about providing children with the skills and the resilience at this stage, made sense to us and seemed to fit with what we know about teaching year five.

Teachers were positive about the programme structure and the way it was organised and felt that the FRIENDS acronym was particularly useful.

I’ve seen each of the things. I’ve worked with the children about how to relax. I’ve worked with the children about their feelings; I’ve worked with the children about helpful thoughts um but never in that order and the order of it really helped it to sink in and helped it to be more successful.

#### Programme Content and Delivery

Over half of the teachers thought that the programme had too many strategies and that there was not enough time to complete them.

……there was so much material in it, it was all really good stuff but that could have been easily a year’s curriculum. Let alone nine sessions…

I think it’s too full, I think there’s too much in it, to try and… every single lesson, I think had too many things to try and get into the time.

They commented that there was too much passive learning, i.e. reading, writing, sitting and listening, but were more positive about the practical exercises and the opportunities the children had to work in groups. Overall, teachers felt that the programme could be improved by making the delivery more practical (less reading and writing) and with more time for discussion.

Where the children can interact with each other more than just listening I think that they were better.

Teachers felt that the session delivery was a little dry and formulaic although were impressed by the logical sequence of the programme and found the session plans useful.

I liked the idea of building something step by step each week, there was very obvious progression within it.

Finally, half of the teachers commented that many of the skills taught within the FRIENDS programme were similar to those taught in their existing PSHE classes. However, despite this overlap, a number commented that because FRIENDS was more focused and explicit in teaching these skills it usefully complimented PSHE and was considered to be a valuable addition.

I think that the FRIENDS programme makes everything more explicit and so it’s… for example dealing with conflict. Yeah, but I think this programme is quite accessible because it was so clear what the issue was that we were discussing. I think, yeah the solutions where actually delivered to the children rather than perhaps with a normal PSHE lesson it kind of comes from them and you all talk about what you do and it comes like that whereas this was more ‘Here’s a good way to do it, try this’. So it was quite useful I think for them.

It is a valuable addition but there is some overlap but I don’t actually mind that. I think these things are good to be done more than once.

#### Programme Structure (Length/Sessions)

Teachers were divided in their views about the length of the programme with just over half reporting that nine was the right amount of sessions.

It was about right for the children, they were happy you know 9, 10 weeks…… it kept their interest, they were looking forward to the lessons all the time, so that was good.

Some suggested merging sessions, feeling that topics could be combined, whereas others suggested less or even more sessions.

There was a similar range of views regarding session length with a slight majority feeling that sessions were too long.

The hour was quite a long time to take out of a whole school week, I must admit, um, a 40 minute session would be probably what you’d want to devote to PSHE…

There were also differing views about how well FRIENDS could practically be fitted within a busy school timetable A few commented that it fitted well, but those who found it hard to make space were equally clear that they did not believe that the programme content should be compromised.

I think it fitted in quite well, I think it was enough time to obviously really, kind of, embed their understanding of like the thoughts and things and um, to obviously talk about previous lessons, so I think it was a reasonable amount of time actually.

It doesn’t, 9 weeks doesn’t fit into a term at all but …… I don’t think there is anything you could take out of the programme to make it, make it fit.

#### FRIENDS Workbook and Resources

Teachers were particularly positive about the FRIENDS workbook with many commenting that children really enjoyed having a workbook.

They took real ownership of their book and I think that was really nice and it’s just something that they can take away with them and look back on if they want to use any of the strategies so that was very positive.

However, the majority thought that there was too much reading and writing in the workbook and that this sometimes distracted children from the lessons.

I think sometimes the children found that the booklet a little bit difficult…they didn’t really express what they were feeling in the booklet. And for some of the children, the actual writing of the booklet was really hard. Just because they found writing hard.

The accompanying resources were very well received, with a couple of teachers suggesting it facilitated learning and made preparation easier and that they would continue to use them.

They were fantastic. [Laughs] And I was so glad that they were there for every week. It just, as a deliverer, it just made my life so much more easy that I didn’t have to worry about it.

#### Most Useful Aspects of the Programme

Teachers felt that the most positive skill the children learned was the ‘Red and Green Thoughts’. This provided children with a way of describing negative and unhelpful ways of thinking which increase anxiety (red thoughts) and positive, helpful ways of thinking which reduce anxiety and encourage the child to face challenges (green thoughts).

I think the red thoughts, green thoughts…… they’ve really taken that on board, I think it was really visual and I think it was really clear to them what they were and they were identifying them…

Relaxation skills were also noted as particularly positive with several teachers positively commenting about the ‘Pizza Massage’ exercise. They noted that the relaxation skills were memorable, quick, promoted useful discussion and provided a useful ways to help the children relax.

Yes, I think that the relaxation part of it, they particularly, the children particularly liked that and I think that….to have a bit of time and to learn some skills on how to de-stress and take yourself out of a challenging situation and give yourself some chill time is really important.

Ways of dealing with problems (e.g. coping step plan and problem-solving techniques) were also identified as helpful with many commenting that it was useful for the children to break problems and challenges down into smaller, achievable steps.

…breaking something down I think was useful, it made them think about how they can break something into smaller chunks, that it’s achievable and it’s a small move to do, rather than quite a big step…

Whilst these two problem solving methods were viewed as useful, some noted that there was a potential for confusion between them.

#### Programme Benefits

Almost all teachers felt the children had benefited from FRIENDS. Many felt the children had been given useful tools and strategies and that the programme had improved the children’s confidence and self-esteem. Others commented that the children had learned to express their feelings, recognise feelings in others and deal with challenging and worrying situations, e.g. preparation for secondary school.

So they’ve got these, kind of, key concepts that are in their heads and they are using them and they are applying them to other lessons……

You could see children’s confidence growing and their belief that they could actually express their ideas and say what they thought and that sort of thing has continued for a lot of them now.

Many teachers felt that the programme had also been beneficial for themselves (the teachers), equipping them with strategies and language, as well learning to understand the children’s feelings.

I …have felt much more empowered. Um, and it’s given me a strategy to know how to help them, rather than just saying ‘oh, sit down, yeah, you’re fine, you’re fine, it’ll be OK’.

#### Continued Use of Skills

Many teachers reported that children continued to use skills particularly the Red and Green Thoughts’ and the relaxation techniques.

I would say it’s the green thoughts and the red thoughts that have had a lasting impact within the class that they’ve actually applied to learning situations.

And encourage them to relax and giving them those extra skills, and also encouraging them to talk to each other, and saying, you know, ‘Are you ok? Do you need to do some Pizza Massage?’ A lot of them were doing that and we still do that now.

However, almost half of the teachers could not identify any particular changes in the children’s behaviour.

Whether or not that’s had any long lasting impact, I think is doubtful, but that’s probably because it isn’t integrated into our curriculum, so we’re not taking it forward…

No, I don’t think there’s been any noticeable difference.

### Parent Views

Interviews were undertaken with a sample of 20 parents, mostly mothers, who were randomly selected from a larger group of 308 who volunteered to take part in detailed interviews for an economic analysis.

#### Overall Impression

Although the majority of parents knew that the FRIENDS programme was running in their child’s school their knowledge about the specific content of the programme was limited. Overall, parents thought that the programme was ‘good’ and that it helped their child become more emotionally aware of themselves and others. Most commented that their child had enjoyed FRIENDS and they appreciated the opportunity to talk about their feelings.

Think it’s really good…I think she understands more about other peoples’ feelings. Including my feelings and other people around her, how she reacts, how it could affect other people…

Well, I think it’s brilliant. I love all the different topics that it covers. I think it’s quite clever in the way it approaches things, it takes sort of everyday situations that all children can relate to, and skills, you know, in terms with how you deal with a situation.

#### Continued Use of Skills

When initially asked parents had not noticed their child using any new skills. However during conversation some parents commented that children had mentioned or used skills and strategies taught during FRIENDS to help them with problem solving and relaxation.

She does use strategies but I don’t know where they’ve come from, for sort of calming down.

He said ‘I liked the step plans… ‘cos it helps you work out how to get somewhere with little steps.

Yes, yeah she does. Before she could be quite hormonal and we would very much flight but now you can see her think, take a breath and she would walk away from a situation or she’ll come and just answer, rather than reacting the way she used to

#### Programme Benefits

Parents noted improvements in the children’s relationships, the ability to talk to peers more easily and described their child as being calmer.

She’s calmer; she seems more in control of her feelings… When she does have a fall out with friends or someone’s nasty to her… She copes with it a lot better now….

Parents also reported positive emotional changes including improvements in mood and increased resilience. All interviewed parents noted an increase in confidence in their child.

His confidence has improved and now he doesn’t take anymore that he has to, he’ll walk away from situations. The other day there was somebody saying something to him and he just went ‘whatever’ and walked away. Whereas before he would have come home and probably burst into tears.

I would say that the project has helped boost her confidence ‘cause I would say that she’s more chatty.

Many parents discussed their child’s increased awareness of their own feelings stating they had developed a deeper understanding of how their emotions can affect them.

She understands that if her tummy feels funny it’s not because she’s sick, but it’s because of butterflies or nervousness, she’s now aware that you get different feelings for different reasons, whereas before she would have said ‘I’ve got a tummy ache, I feel sick’, now she’s thinking about it a bit more, it’s made her much more aware of herself.

Finally, some parents noted that their child had made academic improvements, mostly within literacy but also in numeracy.

Her literacy is definitely better, her writing better than it has been.

Although parents reported positive changes in a range of areas they did not necessarily attribute these to FRIENDS.

I’d sort of put it down to hormones.

I don’t know whether it is more of a maturing thing

## Discussion

Results from a large UK implementation trial found that FRIENDS, provided as a universal intervention in primary schools, was effective in reducing anxiety in children but only when delivered by health staff (Stallard et al., 2014). Whilst this is encouraging, interventions found to be effective are not routinely adopted by schools (Giesen, Searle, & Sawyer, 2007). A range of factors will affect the uptake of an effective programme including the social acceptability and perceived value of the intervention. This paper reports a qualitative analysis of a universal anxiety prevention programme FRIENDS and, to our knowledge, is the first study to report the views of children, school staff and parents.

Overall feedback was positive with children enjoying FRIENDS and teachers feeling that it provided children with useful skills. Children and teachers particularly liked the active hands-on practical activities and group work (role play, scenarios, games etc.) but felt that there was too much passive learning (reading, writing, listening). Although children were fond of the accompanying FRIENDS workbook, especially the more visual elements, teachers felt that the amount of reading and writing was not helpful. This may reflect the type of teaching style that is now common amongst primary schools in England, which involves more active, ‘getting up and doing’, learning.

In terms of the underlying cognitive behaviour therapy (CBT) model, teachers praised the logical and sequential structure which allowed the gradual introduction of new skills. Skills from all three core elements of the CBT model (e.g. cognitive, emotional and behavioural) were noted. Children particularly commented on the behavioural (coping step plan and problem solving) and emotional (relaxation techniques) elements whereas teachers were particularly positive about the cognitive (red and green thoughts) and emotional (relaxation techniques) elements. Whilst many teachers felt that the skills being taught in FRIENDS were similar to those children would be learning through their usual classes, a number felt that FRIENDS provided a more focused and structured approach that complimented this well.

Neither children nor teachers felt there was enough time to cover all of the content during the time available each week. Overall, children wanted more time for FRIENDS as they often felt they were missing out on some of the tasks in the workbook. Teachers were divided, with just over half feeling that the total number of sessions was about right and in terms of session length, approximately half felt that a 45- to 60-min session was too long. There was, however, no consensus amongst the teachers as to whether the programme and sessions should be longer or shorter. This highlights the difficulty of ensuring that emotional health programmes can be accommodated within busy school timetables. If programmes are to be sustainable, they need to be flexible in order to fit within the limited time available in schools. However, there is a tension between flexibility and ensuring that programme fidelity is maintained. Empirically supported programmes such as FRIENDS are based on proven theoretical models (i.e. CBT) which inform programme content. Whilst there may be opportunities for flexibility in the way that sessions are delivered and techniques are introduced, altering programme content and length may compromise effectiveness.

Children felt that FRIENDS had benefited them in many ways and provided a number of examples of ongoing skills usage. In particular, children felt that FRIENDS had helped them with awareness and management of emotions in both themselves and others and felt that relationships with others had improved. They gave examples of how they had used coping step plans to face challenges such as marital arts grading, swimming, tree climbing and school performances. They noted how they used relaxation techniques, e.g. deep breathing, or removing themselves from a situation instead of retaliating resulted in fewer arguments and improvements in relationships. However, whilst children reported continued use of skills teachers did not note any significant changes in overall behaviour in the classroom and parents mainly attributed changes to the child’s increasing age and maturity not FRIENDS. These observations are consistent with our main trial results, where the positive effect of FRIENDS was specific to child report (Stallard et al., 2014). This finding may therefore reflect difficulties in assessing changes in internal states such as anxiety and in cognitive, emotional and problem solving processes which are not directly observable to others.

Our data indicate that there may be some vicarious effects of FRIENDS whereby siblings, peers, parents and teachers, have benefited. Children from around half the schools interviewed reported sharing skills with family and friends, e.g. relaxation, problem-solving and thought challenging, and found these useful. Teachers from a similar number of schools commented that they had found the programme beneficial for themselves. They reported having learnt a lot about emotional health (language and skills) for their own personal development, as well as gaining more emotional insight into the children. This ‘ripple effect’ suggests that universal prevention programmes not only benefit the individual who directly receives them but also those who they share their skills with, and those who deliver the programme.

The results of this study raise important considerations for implementing empirically supported prevention programmes in schools. Whilst FRIENDS, delivered by health staff, had a positive effect on child reported symptoms of anxiety, it will not be widely implemented unless it is supported and seen as important by school staff. This study has highlighted that school staff were critical of the delivery style which they thought was dry and formulaic with too much passive learning. They noted that the programme content overlapped with the current curriculum and were concerned about the additional time involved in delivering FRIENDS. Furthermore, almost half were unable to identify any tangible changes in the children’s behaviour. Given the limited number of identified benefits, concerns about delivery, overlap with current curriculum and limited time within a busy timetable it is unlikely that implementing FRIENDS will be a priority for these schools. Assessing the social validity of programmes from the perspective of different stakeholders will help to identify issues and barriers that mighty influence the adoption and sustainability of empirically supported programmes.

These findings also highlight future areas of research. Firstly, it is important to assess both the clinical effectiveness and social validity of prevention programmes in order to understand factors that might affect their sustainability in schools. A clinically effective programme that does not have ownership from school staff or is not seen as important will not be implemented. Secondly, involving school staff as co-producers of emotional health prevention programmes may help to address concerns about delivery. The teacher’s expert knowledge about learning styles and presenting materials in an engaging way would compliment the expertise of mental health workers about programme content. Finally, programmes should assess a range of outcomes other than changes in mental health status. These might be more tangible and important to school staff such as educational achievement, absenteeism, bullying and attitude to learning. This would go some way to ensuring that empirically supported programmes can be scaled up and sustained in schools (Elias, Zins, Graczyk, Weissberg 2003).

This is the first qualitative evaluation of FRIENDS to assess the views of children, teachers and parents about universally delivered FRIENDS. Data from 182 children, teachers and parents were obtained allowing saturation of dominant themes to emerge. We used a robust qualitative methodology for data collection, analysis, and reporting.

However, our study does have limitations. Firstly, the health-care staff who delivered FRIENDS were not interviewed. Our main trial findings indicate that FRIENDS delivered by health-led staff was more effective than delivery by school staff (Stallard et al., 2014). The views of the health leaders could have provided a valuable perspective on programme delivery and an insight into why these two methods of delivery resulted in different outcomes. Similarly, our analysis was undertaken at a programme level and we did not compare the social validity of children who received health- or school-led FRIENDS. Children may have identified some important differences about the delivery which might contribute to the effectiveness of the programme. Secondly, the schools who participated in our trial were less socially disadvantaged and had more white British participants than the average UK-based school. It is therefore unclear whether similar views would be obtained with a more disadvantaged and ethnically diverse population. Thirdly, the children who took part in our focus groups were self-selected and may be more vocal, confident and positively disposed to FRIENDS. Similarly, parents were also selected from a sub-group of more engaged parents who volunteered to be interviewed and their views may be different from the wider population.

Despite these limitations our findings suggest that participating children, parents and teachers were positive about FRIENDS with children enjoying the programme, learning useful skills and applying them to help themselves and others. These findings are consistent with existing research which suggest that enhancement of a child’s ability to regulate emotions may contribute to resilience and subsequently lead to improved behaviour, relationships, self-esteem and potentially reduce symptoms of anxiety (Ballespi, Jane, & Riba, 2012). Even though no statistically significant effect of FRIENDS was observed on questionnaires assessing the secondary outcomes of self-esteem and worries (Stallard et al., 2014), this qualitative analysis indicates that there were nonetheless positive changes in these areas, as commonly noted by teachers and parents increase in children confidence and self-esteem. Further research is required to establish the social acceptability of FRIENDS with more diverse populations and to establish whether these benefits persist over time. In addition further qualitative studies may help to understand why the same programme can result in different outcomes depending on who delivers it.
